# Prognostic Value of HPV E6/E7 mRNA Assay in Women with Negative Colposcopy or CIN1 Histology Result: A Follow-Up Study

**DOI:** 10.1371/journal.pone.0057600

**Published:** 2013-02-27

**Authors:** Paolo Giorgi Rossi, Maria Benevolo, Amina Vocaturo, Donatella Caraceni, Lucia Ciccocioppo, Antonio Frega, Irene Terrenato, Roberta Zappacosta, Deborah French, Sandra Rosini

**Affiliations:** 1 Agency for Public Health Lazio Sanità, Rome, Italy; Epidemiology Unit, AUSL Reggio Emilia, Reggio Emilia, Italy; 2 Pathology Department, Regina Elena National Cancer Institute, Rome, Italy; 3 Cytopathology Unit, “F. Renzetti” Hospital, Lanciano, Chieti, Italy; 4 Gynecology Department, 2nd Medicine Faculty of “La Sapienza” University, “Sant’Andrea” Hospital, Rome, Italy; 5 Epidemiology Department, Regina Elena National Cancer Institute, Rome, Italy; 6 Oncology and Neurosciences Department, Cytopathology Section, “G. D’Annunzio” University, Chieti, Pescara, Italy; 7 Clinical and Molecular Medicine Department, 2nd Medicine Faculty of “La Sapienza” University, “Sant’Andrea” Hospital, Rome, Italy; Queensland Institute of Medical Research, Australia

## Abstract

Pap test, and especially HPV DNA test, identify a large group of women who do not have any clinically relevant lesions, i.e., CIN2+ (Cervical Intraepithelial Neoplasia grade 2 or worse), but who are at greater risk of getting lesions in the future. The follow up of these women needs new biomarkers with prognostic value. The objective of this study is to evaluate the prognostic value of E6/E7 mRNA over-expression assay (PreTect HPV-Proofer, Norchip) for 5 HR-HPV types (16, 18, 31, 33, and 45) for progression to CIN2+ after a negative colposcopy. This prospective study, conducted at four Italian centres, enrolled 673 women with either a negative colposcopy or a negative or CIN1 histology. The clinical end-point was histological confirmation of CIN2+. Women were classified at baseline according to mRNA results and managed according to local colposcopy protocols. At least one conclusive follow-up test was obtained for 347 women (25 months average lapse since recruitment, range 5–74). Only seven CIN2+ were detected during follow up, three among the 82 women positive for mRNA at baseline, two among the 250 negative (Fisher exact test, p = 0.02), and two among the 12 with an invalid test. Absolute CIN2+ risk was 6.7/1,000 person/years in the whole cohort. The absolute CIN2+ risk was 18.4/1,000 person/years and 3.6/1,000 person/years in mRNA-positive and mRNA-negative women, respectively. In conclusion, E6/E7 mRNA over-expression appears to be a good candidate as a prognostic biomarker to manage HR-HPV DNA-positive women with negative colposcopy or histology, particularly in order to decrease follow-up intensity in those who are negative.

## Introduction

Cervical cancer screening is one of the most effective interventions ever implemented in medicine [1]. The ability of the Pap test, and now of the HPV DNA test, to identify women at risk of precancerous lesions and the availability of effective and relatively non-invasive treatment for these lesions has allowed us to control the incidence and mortality for cervical cancer in most industrialised countries [2], [3]. Many women get abnormal Pap test results in cervical cancer screening [4], [5] and many more will test HPV-positive [6]–[10] when HPV is used as primary screening test, although only a few of them will have precancerous lesions, i.e. a Cervical Intraepithelial Neoplasia grade 2 or 3 (CIN2 or 3). While many of these women will require a colposcopy, and many of these colposcopies will not find any high-grade lesion, these women are nevertheless at greater risk of having or developing a CIN2 or 3 for a certain amount of time [11], [12]. This is due both to the low sensitivity of colposcopy [13]–[18] and to the progression of the HPV infection from negative histology or CIN1 to more clinically relevant lesions [19], [20].

These women are difficult to manage since infections and cytological modifications can take a long time to clear and regress [12], [21]. At present, these women are usually referred to a new colposcopy or to cytology at 6- or 12-month intervals until Pap test or HPV DNA are negative.

Biomarkers are needed to identify those HPV infections at risk of developing into a high-grade lesion in a short amount of time, i.e., less than the normal screening interval. The most promising targets for such novel biomarkers are the E6/E7 viral oncogene expression and its molecular consequences [22]–[24]. HPV oncogene active transcription and its effects on the host cell can be monitored directly through the detection of E6/E7 viral mRNA transcripts [25]–[28], or indirectly, for example through the detection of the overexpression of the host protein p16 [29]–[32].

Commercially available robust assays for HPV mRNA detection can be performed by reflex after liquid-based cytology or HPV-DNA test [27], [32]–[41].

In this study, we measured the prognostic value – the ability to identify women who will develop CIN2 or 3 during follow up – of the PreTect HPV-Proofer E6/E7 mRNA assay (Norchip) in women with negative colposcopy or CIN1 histology.

## Materials and Methods

### Setting

This prospective study collected data from four Italian gynaecological prevention clinics: a research hospital (Regina Elena National Cancer Institute of Rome), two teaching hospitals (the “G. D’Annunzio” University of Chieti, and the “S. Andrea” Hospital, affiliated with the “La Sapienza” University of Rome), and one cervical cancer screening centre (the “F. Renzetti” Hospital of Lanciano Vasto). All are public institutions and virtually all the tests are paid for by the National Health Care System. The Regina Elena National Cancer Institute Ethics Committee confirmed that the study did not require IRB approval, only IRB notification, based on Italian legislation (determination AIFA of the 20th of March 2008, section 10). Patient consent was not required, based on the Italian personal data protection code (legislative decree no. 196 dated June 30 2003). The patients were managed strictly following the clinical indications related to their pathology and according to the National Guide Lines and good clinical practice. They did not receive different care on the basis of study findings and study results did not affect patient treatments. All data was analyzed anonymously.

### Patients

We recruited all patients who met the following inclusion criteria: they had an mRNA test; they were > = 18 years of age; they had a colposcopic assessment within five months from mRNA test, with negative result without biopsy or a negative or CIN1 biopsy. Women were referred for colposcopy because of an abnormal Pap test or a positive HPV test.

Exclusion criteria were: treatment for cervical lesions in the previous five years or a history of any type of cancer; having a CIN2 or more severe cervical neoplasia (CIN2+) at baseline colposcopy-guided biopsy; undergoing a surgical or ablative treatment during baseline colposcopy.

Enrolment lasted from January 2004 to December 2006. All subjects were followed up until 30 June 2011. Recruitment started from the date of collection of the cervical sample for the mRNA test. Tests performed more than 5 months after baseline assessment colposcopy were considered as follow-up tests in order to rule out that repeated colposcopies were due to technical reasons and to be sure of not including prevalent lesions.

### Follow up

Follow up was performed according to local colposcopy protocols. Negativity for CIN2+ at a given time was defined by a negative cytology or a negative HR-HPV DNA test. In the event of ASC-US or more severe cytology or a positive HR-HPV DNA test, being negative for CIN2+ was determined by means of a negative colposcopy or a colposcopy-guided biopsy that was histologically negative or CIN1. When the last follow up episode is a positive cytology or HPV not followed by colposcopy, the episode was considered not conclusive and the follow up was censored at the last conclusive episode. Women were considered positive for CIN2+ only with a histology-confirmed result. Women with surgical or ablative treatment, without histological confirmation of CIN2 or more severe diagnosis, were censored.

### Cytological Diagnoses

Cervical samples were taken by cytobrush (Hologic, Rome, Italy) and plastic Ayre’s spatula (Hologic) according to the manufacturer’s instructions and stored in 20 ml of PreservCyt solution (Hologic) at 4°C until use. Liquid-based cytology was prepared using the ThinPrep 2000 System following the manufacturer’s instructions (Hologic). The cytological specimens were reported using the 2001 Bethesda reporting system [42].

### E6/E7 mRNA Detection

Five ml of PreservCyt solution were used for the detection of E6/E7 mRNA of HPV types 16, 18, 31, 33, and 45 within 14 days of sample collection by means of the PreTect HPV-Proofer Kit (referred to as the mRNA test) (Norchip, Klokkarstua, Norway) according to the manufacturer’s instructions. mRNA was extracted using the RNeasy Mini Kit (QIAGEN, Italy). The PreTect HPV-Proofer utilizes an isothermal nucleic acid sequence-based amplification (NASBA), which amplifies mRNA in a DNA background, detecting and genotyping HPV transcripts in the same reaction. The amplified products were detected in real time using fluorescent-labelled molecular beacon probes directed against full-length E6/E7 mRNA. Accumulated mRNA fluorescent profiles were analyzed and ascribed positive/negative status, for each type included, by the supplied PreTect analysis software. Human U1 small ribonucleoprotein (U1A mRNA) was used as an RNA integrity/adequacy internal control. When the U1A amplification was not detected, the test result was qualified as invalid.

### HR-HPV DNA Testing

Testing for HR-HPV DNA was performed by the HC2 test (QIAGEN), a semi-quantitative signal-amplified hybridization assay for the chemiluminescent detection of the 13 most common HR-HPV types (16, 18, 31, 33, 35, 39, 45, 51, 52, 56, 58, 59, and 68), according to manufacturer’s instructions. Before performing the HC2 test, 4 ml of PreservCyt solution were processed with the HC2 Sample Conversion Kit (QIAGEN). The positive cut-off (CO) value was considered the mean of the positive control samples. The results were considered positive when the ratio between the relative-light units of the sample (RLU) and the chosen positive CO (RLU/CO) was 1.00 or higher. The repeat zone between 1.00 and 2.50 was not used.

### Statistical Analysis

All statistical analyses were performed with STATA software version 11. The difference in CIN2+ incidence between positives and negatives to the mRNA test at baseline, as well as progression to CIN1 and CIN1 persistence, were calculated with the Log Rank test.

## Results

The mean age of the enrolled women was 35.6 years (SD = 10.0); the range was 18–71 years, with 96% between 21 and 65 years. Table 1 shows the baseline characteristics of the 673 subjects. Three hundred forty-seven enrolled women had at least one follow-up test (51.6%). Mean follow-up time was 25.2 months, ranging from 5 to 74.4 months. The average number of follow-up tests was 2.1, 1.3, 0.62, and 0.43 for cytology, HR-HPV DNA, colposcopy and histology, respectively.

**Table 1:** Baseline characteristics and presence of follow up among the 673 enrolled women stratified by mRNA test result.

		mRNA test							
		Negative		Positive		Invalid		Total	
		N (row%) (col%)		N (row%) (col%)		N (row%) (col%)		N (row%) (col%)	
**Total**		496 (73.7) (100)		150 (22.3) (100)		27 (4.0) (100)		673 (100) (100)	
**HR-HPV DNA**									
Negative		160 (98.2) (32.3)		3 (1.8) (2.0)		0 (0.0) (0.0)		163 (100) (24.2)	
Positive		329 (65.8) (66.3)		144 (28.8) (96.0)		27 (5.4) (100)		500 (100) (74.3)	
Not available		7 (70.0) (1.4)		3 (30.0) (2.0)		0 (0.0) (0.0)		10 (100) (1.5)	
**Cytology**									
NILM		137 (79.7) (27.6)		25 (14.5) (16.7)		10 (5.8) (37.0)		172 (100) (25.6)	
ASC-US		93 (73.8) (18.8)		26 (20.6) (17.2)		7 (5.6) (26.0)		126 (100) (18.7)	
L-SIL		244 (72.0) (49.2)		85 (25.1) (56.7)		10 (2.9) (37.0)		339 (100) (50.4)	
ASC-H/H-SIL		14 (51.8) (2.8)		13 (48.2) (8.7)		0 (0.0) (0.0)		27 (100) (4.0)	
Not available		8 (88.9) (1.6)		1 (11.1) (0.7)		0 (0.0) (0.0)		9 (100) (1.3)	
**Histology**									
no biopsy (negative colposcopy)		137 (77.0) (27.6)		30 (16.8) (20.0)		11 (6.2) (40.8)		178 (100) (26.5)	
Unsatisfactory		0 (0.0) (0.0)		1 (100) (0.7)		0 (0.0) (0.0)		1 (100) (0.1)	
negative biopsy		110 (76.4) (22.2)		28 (19.4) (18.7)		6 (4.2) (22.2)		144 (100) (21.4)	
CIN1		249 (71.1) (50.2)		91 (26.0) (60.6)		10 (2.9) (37.0)		350 (100) (52.0)	
**presence of FU test**									
yes	250 (72.1) (50.4)		82 (23.6) (54.6)		15 (4.3) (55.6)		347 (100) (51.6)		
not	246 (75.4) (49.6)		68 (20.9) (45.4)		12 (3.7) (44.4)		326 (100) (48.4)		

One hundred sixty-three (24.6%) women were negative for HR-HPV DNA, only three of whom had a positive mRNA test. No CIN2+ occurred in the 81 who had at least one follow-up test.

During follow up, only 7 high-grade CIN, five CIN2, and two CIN3 (Tables 2, 3, 4, and 5) were identified. The absolute risk was 6.7/1,000 person/years (95% CI 2–16) in the whole cohort. Three CIN2+ occurred in women who were mRNA positive at baseline, two in women mRNA negative at baseline, while the other 2 CIN2+ occurred in women with invalid mRNA result at baseline (Table 2). The absolute risk among mRNA positives was 18.4/1,000 person/years (95% CI 4–53) compared to 3.7/1,000 person/years (95% CI 0.4–13) of the mRNA negatives, while the incidence rate ratio was 5.0 (95% CI 0.78–26.9; log-rank test for equality of survival time p = 0.049). Since no CIN2+ occurred in women HR-HPV DNA-negative at baseline, the absolute risk in HR-HPV DNA-negative women was 0 in comparison with 11.2/1,000 person/years in the HR-HPV DNA-positive women (95% CI 4–24; log-rank test for equality of survival time in HR-HPV DNA positive *vs* HR-HPV DNA negative p = 0.152) (Table 3).

**Table 2:** Follow up histology results for women with at least one follow up test by mRNA test result. at baseline.

	mRNA test at baseline							
Follow up results	Negative		Positive		Invalid		Total	
	N (row%) (col%)		N (row%) (col%)		N (row%) (col%)		N (row%) (col%)	
Total	250 (72.1) (100)		82 (23.6) (100)		15 (4.3) (100)		347 (100) (100)	
No biopsy	206		63		11		280	
	(82.4)		(76.8)		(73.4)		(80.7)	
CIN1	42		16		2		60	
	(16.8)		(19.5)		(13.3)		(17.3)	
CIN2+	2		3		2		7	
	(0.8)		(3.7)		(13.3)		(2.0)	
*Absolute risk* *	*3.7*		*18.4*		*57.5*		*6.7*	
*95%CI*	*0.4–13*	*4–53*		*7–192*		*2–16*		

* CIN2+/1,000 person/years.

**Table 3:** Follow up histology results for women with at least one follow up test by HR-HPV DNA result at baseline.

	HR-HPV DNA test at baseline			
Follow up results	Negative	Positive	Not available	Total
	N (row%) (col%)	N (row%) (col%)	N (row%) (col%)	N (row%) (col%)
Total	81 (23.3) (100)	257 (74.1) (100)	9 (2.6) (100)	347 (100) (100)
No biopsy	68	204	8	280
	(84.0)	(79.4)	(88.9)	(80.7)
CIN1	13	47	0	60
	(16)	(18.3)	(0)	(17.3)
CIN2+	0	6	1	7
	(0)	(2.3)	(11.1)	(2.0)
*Absolute risk* *	*0*	*11.2*		*6.7*
*95%CI*	*0–16*	*4–24*		*2–16*

* CIN2+/1,000 person/years.

**Table 4:** Follow up histology results for women with at least one follow up by cytology at baseline.

Follow up results	NILM	ASC-US	L-SIL	ASC-H/H-SIL	Not available	Total
	N (row%) (col%)	N (row%) (col%)	N (row%) (col%)	N (row%) (col%)	N (row%) (col%)	N (row%) (col%)
Total	76 (21.9) (100)	67 (19.3) (100)	184 (53.0) (100)	16 (4.6) (100)	4 (1.2) (100)	347 (100) (100)
No biopsy	60	57	149	10	4	280
	(79.0)	(85.1)	(81.0)	(62.6)	(100)	(80.7)
CIN1	14	10	33	3	0	60
	(18.4)	(14.9)	(17.9)	(18.7)	(0)	(17.3)
CIN2+	2	0	2	3	0	7
	(2.6)	(0)	(1.1)	(18.7)	(0)	(2.0)
*Absolute risk* *	*11.7*	*3.7*		*97.7*		*6.7*
*95%CI*	*1–42*	*0.5–13*		*20–257*		*2–16*

* CIN2+/1,000 person/years.

**Table 5:** Follow up histology results for women with at least one follow up by histology at baseline.

	Histology at baseline			
Follow up results	No Biopsy	Negative	CIN1	Total
	N (row%) (col%)	N (row%) (col%)	N (row%) (col%)	N (row%) (col%)
Total	89 (25.7) (100)	66 (19.0) (100)	192 (55.3) (100)	347 (100) (100)
No biopsy	79	57	144	280
	(88.8)	(86.3)	(75.0)	(80.7)
CIN1	9	5	46	60
	(10.1)	(7.6)	(24.0)	(17.3)
CIN2+	1	4	2	7
	(1.1)	(6.1)	(1.0)	(2.0)
*Absolute risk* *	*5.0*	*26.3*	*5.1*	*6.7*
*95%CI*	*0.1–27*	*7–66*	*0.6–18*	*2–16*

* CIN2+/1,000 person/years.

The 7 CIN2+ had the following cytological results at baseline: two Negative for Intraepithelial Lesion or Malignancy (NILM), two Low Grade Squamous Intraepithelial Lesions (L-SIL), one Atypical Squamous Cells cannot exclude High Grade Squamous Intraepithelial Lesion (ASC-H), and two High Grade Squamous Intraepithelial Lesions (H-SIL) (Table 4). Baseline histology was four negative and two CIN1, while one case had no biopsy (Table 5). There was no difference in risk for NILM compared to ASC-US or L-SIL cytology (absolute risk 11.7, 95% CI 1–42, *vs* 3.7, 95% CI 0.5–13), although ASC-H or H-SIL cytology significantly predicted the risk of CIN2+ (absolute risk 97.7, 95% CI 20–257).

Table 6 shows the distribution of the genotypes identified among the 82 mRNA positive women who were followed up, stratified by follow-up histology results. Three cases of co-infection with two genotypes were observed among the 63 patients with no biopsy during follow up. Of the three mRNA-positive cases that developed a CIN2+ lesion, two were positive for HPV16 and one for HPV18.

**Table 6:** Distribution of the genotypes among the 82 mRNA positive women with at least one follow up test stratified by follow up histology results.

	HPV genotype				
Follow up results	HPV 16	HPV 18	HPV 31	HPV 33	HPV 45
	N (row%) (col%)	N (row%) (col%)	N (row%) (col%)	N (row%) (col%)	N (row%) (col%)
Total	37 (43.5) (100)	11 (11.9) (100)	5 (5.8) (100)	16 (18.8) (100)	16 (18.8) (100)
No biopsy	28	10	1	13	14
	(75.7)	(90.9)	(20.0)	(81.2)	(87.5)
CIN1	7	0	4	3	2
	(18.9)	(0)	(80.0)	(18.8)	(12.5)
CIN2+	2	1	0	0	0
	(5.4)	(9.1)	(0)	(0)	(0)

Moreover, as shown in Tables 7 and 8, there were no differences in progression from negative to CIN1 (8.8% and 9.1% in mRNA negative and positive cases, respectively), nor in CIN1 persistence (23.4% and 27.0% in mRNA negative and -positive samples, respectively).

**Table 7:** Follow up histology results of the women with histology results at baseline by negativity of mRNA test.

	Histology at baseline			
Follow up results	No Biopsy (Negative colposcopy)	Negative Biopsy	CIN1	Total
	N (row%) (col%)	N (row%) (col%)	N (row%) (col%)	N (row%) (col%)
Total	68 (27.2) (100)	45 (18.0) (100)	137 (54.8) (100)	250 (100) (100)
No biopsy	62	40	104	206
	(91.2)	(88.9)	(75.9)	(82.4)
CIN1	6	4	32	42
	(8.8)	(8.9)	(23.4)	(16.8)
CIN2+	0	1	1	2
	(0)	(2.2)	(0.7)	(0.8)

Progression to CIN1 = 8.8% (95% CI 4–16).

CIN1 persistence  = 23.4% (95% CI 17–31).

**Table 8:** Follow up histology results of the women with histology results at baseline by positivity of mRNA test.

	Histology at baseline			
Follow up results	No biopsy (Negative colposcopy)	Negative biopsy	CIN1	Total
	N (row%) (col%)	N (row%) (col%)	N (row%) (col%)	N (row%) (col%)
Total	15 (18.3) (100)	18 (22.0) (100)	49 (59.8) (100)	82 (100) (100)
No biopsy	13	14	36	63
	(86.7)	(77.8)	(73.5)	(76.8)
CIN1	2	1	13	16
	(13.3)	(5.6)	(26.5)	(19.5)
CIN2+	0	3	0	2
	(0)	(16.7)	(0)	(2.4)

Progression to CIN1 = 9.1% (95% CI 2–24).

CIN1 persistence  = 27.0% (95% CI 15–41).

## Discussion

Although the association between E6/E7 mRNA overexpression and CIN severity has been demonstrated in several studies [25]–[27], [32], [36], this is one of the first studies with long follow up of women tested with mRNA and with negative colposcopy or histological results. Our results suggest that E6/E7 mRNA positivity for the five HPV oncogenic types may have a relevant prognostic value for cervical precancer, even if the small number of CIN2+ detected makes the difference only borderline statistically significant and chance might influence our results.

The risk of developing a CIN2+ is five times higher in mRNA-positive than in mRNA-negative women. The latter make up about three quarters of our sample and have a risk of 3.7/1,000 person/year, only slightly higher than those in the general population [5]. This information could be used to modulate the follow up after a negative colposcopy or CIN1 histology, if the mRNA test is performed on the specimens collected immediately before colposcopy.

H-SIL and ASC-H cytology identified a small group of women at very high risk, whereas there were no differences between negative and ASC-US or L-SIL cytology.

It must be noted that HR-HPV DNA test is more sensitive than mRNA test and no CIN2+ occurred in the two-year average follow up of the 81 HR-HPV DNA-negative women. Most of the guidelines recommend that the HR-HPV DNA-negative women with negative or CIN1 colposcopy should be referred to normal screening interval [11], [43], with few exceptions. Furthermore, more recent guidelines [43], [44] generally do not recommend colposcopy for HR-HPV DNA-negative women, unless they have high grade cytology (ASC-H/HSIL). Consequently, in the near future it will no longer make sense to use HR-HPV DNA test to modulate the management of women with negative colposcopy, given that almost all the women referred to colposcopy will be HR-HPV DNA-positive.

A lower sensitivity and higher specificity of PreTect HPV-Proofer assay compared to HPV-DNA tests and to other E6–E7 mRNA tests, have already been reported [36], [40]. A high specificity and a low positivity rate of a HPV mRNA test meanslow referral rate for colposcopy which is very appealing in a triage setting [39], [41], [45]. Furthermore, sensitivity which is not too close to 100% is a necessary, but not sufficient condition, for a prognostic biomarker able to distinguish between progressive and regressive CIN2 [46]. However, further research is needed to understand whether or not we can use any biomarker in order to decide not to treat a CIN2.

It must be highlighted that two CIN2+ lesions occurred during the follow up of the 27 women with invalid mRNA result, making this group at high risk. Previous studies observed that women with invalid mRNA result were not a random sample of the average tested population [27], although an increased risk for CIN2+ was never observed. These conflicting results make more evident the need for retesting women with invalid HPV mRNA tests.

Neither the progression from negative to CIN1 histology nor the persistence of CIN1 was associated with mRNA positivity at baseline. This observation, even though limited by the very low statistical power of this study, confirms the idea that CIN1 should not be considered as a screening target, it should not be treated [11] and it has no prognostic value [20], [47].

Despite the relatively high number of eligible subjects, we had a high proportion of loss to follow up and very low risk of progression to CIN2+ in HR-HPV DNA-positive women compared to what has been found in other cohort studies [19], [48]. Two cohort studies that tested cervical dysplasia biomarkers (E6/E7 mRNA and p16 overexpression) [47], [49] observed that about 25% of the subjects with CIN1 histology worsened. However, as the follow up in these studies included assessments occurring very soon after recruitment, a certain amount of contamination from prevalent lesions cannot be ruled out: in the ALTS study it has been estimated that most CIN3 cases diagnosed within two years were prevalent cases [18].

There are many possible explanations for the low risk of progression found in our cohort: 1) the study subjects came from a highly screened population, with very low prevalence of high grade lesions; 2) during follow up, in our study the colposcopy and biopsy rate were quite low compared to previous studies [47], [49]; 3) some CIN1 may have been treated outside of the study with ablative outpatient surgery, diminishing the probability of progression; 4) finally, we tried to reduce contamination from prevalent lesions using very strict criteria in our definition of baseline assessments. It is less probable that true CIN2+ were treated outside of the study and not registered in the clinical records On the other hand, colposcopy of HR-HPV DNA-positive women or histology-negative subjects in the NTCC study had a 2.2% cumulative incidence in 3.5 years, i.e., about 6/1,000/year [50].

### Conclusions

High-grade cytology (H-SIL and ASC-H) is still the strongest predictor of developing a colposcopically detectable CIN2+ and the follow up after negative colposcopy should be intensive for women with this cytology. On the other hand, in well-established screening programmes, women with low-grade cytology may be retested after longer intervals than currently performed. E6/E7 mRNA overexpression for HPV types 16,18, 31, 33, and 45 seems to be a good candidate as a prognostic biomarker to determine the intensity of follow up in HR-HPV DNA-positive women after a negative colposcopy or histology, even though more research is needed.

## References

[1] IARC (2005) IARC Handbooks of Cancer Prevention. Vol. 10. Cervix Cancer Screening. IARC, Lion.

[2] Cuzick J, Mayrand MH, Ronco G, Snijders P, Wardle J (2006) Chapter 10: New dimensions in cervical cancer screening. Vaccine 24 Suppl 3S3/90–7. 10.1016/j.vaccine.2006.05.122 16950022

[3] Giorgi Rossi P, Ricciardi A, Cohet C, Palazzo F, Furnari G, et al (2009) Epidemiology and costs of cervical cancer screening and cervical dysplasia in Italy. BMC Public Health 9: 71. 19243586 10.1186/1471-2458-9-71 PMC2651166

[4] The NHS Information Centre, Public Health Indicators and Population Statistics Team Cervical Screening Programme England (2010) Available: http://www.ic.nhs.uk/webfiles/publications/008_Screening/cervscreen0910/2009_10_Cervical_Bulletin_Final_Report_AI_v1F.pdf.Accessed 2011 Jul 31.

[5] Ronco G, Giubilato P, Naldoni C, Zorzi M, Anghinoni E, et al (2010) Extension of organised cervical cancer screening programmes in Italy and their process indicators: 2008 activity. Epidemiol Prev 34 (Suppl 4)35–51. 21220836

[6] Anttila A, Kotaniemi-Talonen L, Leinonen M, Hakama M, Laurila P, et al (2010) Rate of cervical cancer, severe intraepithelial neoplasia, and adenocarcinoma in situ in primary HPV DNA screening with cytology triage: randomised study within organised screening programme. BMJ Apr 24 340: c1804 doi:10.1136/bmj.c1804. 10.1136/bmj.c1804 PMC3191726 20423964

[7] Bulkmans NW, Berkhof J, Rozendaal L, van Kemenade FJ, Boeke AJ, et al (2007) Human papillomavirus DNA testing for the detection of cervical intraepithelial neoplasia grade 3 and cancer: 5-year follow-up of a randomised controlled implementation trial. Lancet 370: 1764–1772. 17919718 10.1016/S0140-6736(07)61450-0

[8] Kitchener HC, Almonte M, Thomson C, Wheeler P, Sargent A, et al (2009) HPV testing in combination with liquid-based cytology in primary cervical screening (ARTISTIC): a randomised controlled trial. Lancet Oncol 10: 672–682. Erratum in: Lancet Oncol Aug 10(8): 748. 10.1016/S1470-2045(09)70156-1 19540162

[9] Naucler P, Ryd W, Tornberg S, Strand A, Wadell G, et al (2007) Human papillomavirus and Papanicolaou tests to screen for cervical cancer. N Engl J Med 357: 1589–1597. 17942872 10.1056/NEJMoa073204

[10] Ronco G, Giorgi Rossi P, Carozzi F, Confortini M, Dalla Palma P, et al (2008) Results at recruitment from a randomized controlled trial comparing human papillomavirus testing alone with conventional cytology as the primary cervical cancer screening test. J Natl Cancer Inst 100: 492–501. 18364502 10.1093/jnci/djn065

[11] Arbyn M, Anttila A, Jordan J, Ronco G, Schenck U, et al.. (2008) European guidelines for quality assurance in cervical cancer screening. 2^nd^ edition. Luxembourg: Office for Official Publications of the European Communities.

[12] Castle PE, Rodríguez AC, Burk RD, Herrero R, Hildesheim A, et al (2009) Neither one-time negative screening tests nor negative colposcopy provides absolute reassurance against cervical cancer. Int J Cancer 125: 1649–1656. 19569231 10.1002/ijc.24525 PMC2766540

[13] Massad LS, Jeronimo J, Katki HA, Schiffman M (2009) National Institutes of Health/American Society for Colposcopy and Cervical Pathology Research Group (2009) The accuracy of colposcopic grading for detection of high-grade cervical intraepithelial neoplasia. J Low Genit Tract Dis 13: 137–144. 19550210 10.1097/LGT.0b013e31819308d4 PMC2921444

[14] Mitchell MF, Schottenfeld D, Tortolero-Luna G, Cantor SB, Richards-Kortum R (1998) Colposcopy for the diagnosis of squamous intraepithelial lesions: a meta-analysis. Obstet. Gynecol 91: 626–631. 10.1016/s0029-7844(98)00006-4 9540955

[15] Pretorius RG, Belinson JL, Burchette RJ, Hu S, Zhang X, et al (2011) Regardless of skill, performing more biopsies increases the sensitivity of colposcopy. J Low Genit Tract Dis 15: 180–188. 21436729 10.1097/LGT.0b013e3181fb4547

[16] Zuchna C, Hager M, Tringler B, Georgoulopoulos A, Ciresa-Koenig A, et al (2010) Diagnostic accuracy of guided cervical biopsies: a prospective multicenter study comparing the histopathology of simultaneous biopsy and cone specimen. Am J Obstet Gynecol 203: 321.e1–6. 20633870 10.1016/j.ajog.2010.05.033

[17] Stoler MH, Vichnin MD, Ferenczy A, Ferris DG, Perez G, et al (2011) The accuracy of colposcopic biopsy: analyses from the placebo arm of the Gardasil clinical trials. Int J Cancer 128: 1354–1362. 20506504 10.1002/ijc.25470

[18] Castle PE, Gravitt PE, Wentzensen N, Schiffman M (2012) A descriptive analysis of prevalent *vs* incident cervical intraepithelial neoplasia grade 3 following minor cytologic abnormalities. Am J Clin Pathol 138: 241–246. 22904136 10.1309/AJCPNTK6G2PXWHOO

[19] Kjaer SK, Frederiksen K, Munk C, Iftner T (2010) Long-term absolute risk of cervical intraepithelial neoplasia grade 3 or worse following human papillomavirus infection: role of persistence. J Natl Cancer Inst 102: 1478–1488. 20841605 10.1093/jnci/djq356 PMC2950170

[20] Castle PE, Gage JC, Wheeler CM, Schiffman M (2011) The clinical meaning of a cervical intraepithelial neoplasia grade 1 biopsy. Obstet Gynecol 118: 1222–1229. 22105250 10.1097/AOG.0b013e318237caf4 PMC3229199

[21] Kitchener HC, Almonte M, Gilham C, Dowie R, Stoykova B, et al.. (2009) ARTISTIC: a randomised trial of human papillomavirus (HPV) testing in primary cervical screening. Health Technol Assess 13: 1–150, iii–iv. 10.3310/hta13510 19891902

[22] Dockter J, Schroder A, Hill C, Guzenski L, Monsonego J, et al (2009) Clinical performance of the APTIMA HPV Assay for the detection of high-risk HPV and high-grade cervical lesions. J Clin Virol 45 (Suppl 1)S55–61. 19651370 10.1016/S1386-6532(09)70009-5

[23] von Knebel Doeberitz M (2002) New markers for cervical dysplasia to visualise the genomic chaos created by aberrant oncogenic papillomavirus infections. Eur J Cancer 38: 2229–2242. 12441259 10.1016/s0959-8049(02)00462-8

[24] Cuschieri K, Wentzensen N (2008) Human papillomavirus mRNA and p16 detection as biomarkers for the improved diagnosis of cervical neoplasia. Cancer Epidemiol Biomarkers Prev 17: 2536–2545. 18842994 10.1158/1055-9965.EPI-08-0306 PMC2900792

[25] Benevolo M, Terrenato I, Mottolese M, Marandino F, Carosi M, et al (2011) Diagnostic and prognostic validity of the human papillomavirus E6/E7 mRNA test in cervical cytological samples of HC2-positive patients. Cancer Causes Control 22: 869–875. 21424209 10.1007/s10552-011-9757-0

[26] Benevolo M, Vocaturo A, Caraceni D, French D, Rosini S, et al (2011) Sensitivity, specificity, and clinical value of human papillomavirus (HPV) E6/E7 mRNA assay as a triage test for cervical cytology and HPV DNA test. J Clin Microbiol 49: 2643–2650. 21525231 10.1128/JCM.02570-10 PMC3147832

[27] Castle PE, Dockter J, Giachetti C, Garcia FA, McCormick MK, et al (2007) A cross-sectional study of a prototype carcinogenic human papillomavirus E6/E7 messenger RNA assay for detection of cervical precancer and cancer. Clin Cancer Res 13: 2599–2605. 17473189 10.1158/1078-0432.CCR-06-2881

[28] Lie AK, Kristensen G (2008) Human papillomavirus E6/E7 mRNA testing as a predictive marker for cervical carcinoma. Expert Rev Mol Diagn 8: 405–415. 18598223 10.1586/14737159.8.4.405

[29] Donà MG, Vocaturo A, Giuliani M, Ronchetti L, Rollo F, et al (2012) p16/Ki-67 dual staining in cervico-vaginal cytology: Correlation with histology, Human Papillomavirus detection and genotyping in women undergoing colposcopy.Gynecol Oncol. 126: 198–202. 10.1016/j.ygyno.2012.05.004 22588180

[30] Carozzi F, Confortini M, Dalla Palma P, Del Mistro A, Gillio-Tos A, et al (2008) Use of p16-INK4A overexpression to increase the specificity of human papillomavirus testing: a nested substudy of the NTCC randomised controlled trial. Lancet Oncol 9: 937–945. 18783988 10.1016/S1470-2045(08)70208-0

[31] Klaes R, Friedrich T, Spitkovsky D, Ridder R, Rudy W, et al (2001) Overexpression of p16(INK4A) as a specific marker for dysplastic and neoplastic epithelial cells of the cervix uteri. Int J Cancer 92: 276–284. 11291057 10.1002/ijc.1174

[32] Szarewski A, Ambroisine L, Cadman L, Austin J, Ho L, et al (2008) Comparison of predictors for high-grade cervical intraepithelial neoplasia in women with abnormal smears. Cancer Epidemiol Biomarkers Prev 17: 3033–3042. 18957520 10.1158/1055-9965.EPI-08-0508

[33] Halfon P, Benmoura D, Agostini A, Khiri H, Martineau A, et al (2010) Relevance of HPV mRNA detection in a population of ASCUS plus women using the NucliSENS EasyQ HPV assay. J Clin Virol 47: 177–181. 20022804 10.1016/j.jcv.2009.11.011

[34] Lie AK, Risberg B, Borge B, Sandstad B, Delabie J, et al (2005) DNA- versus RNA-based methods for human papillomavirus detection in cervical neoplasia. Gynecol Oncol 97: 908–915. 15943992 10.1016/j.ygyno.2005.02.026

[35] Molden T, Nygard JF, Kraus I, Karlsen F, Nygard M, et al (2005) Predicting CIN2+ when detecting HPV mRNA and DNA by PreTect HPV-proofer and consensus PCR: A 2-year follow-up of women with ASCUS or LSIL Pap smear. Int J Cancer 114: 973–976. 15645423 10.1002/ijc.20839

[36] Ratnam S, Coutlee F, Fontaine D, Bentley J, Escott N, et al (2010) Clinical performance of the PreTect HPV-Proofer E6/E7 mRNA assay in comparison with that of the Hybrid Capture 2 test for identification of women at risk of cervical cancer. J Clin Microbiol 48: 2779–2785. 20573862 10.1128/JCM.00382-10 PMC2916571

[37] Rosini S, Zappacosta R, Di Bonaventura G, Caraceni D, Pilla D, et al (2007) Management and triage of women with human papillomavirus infection in follow-up for low-grade cervical disease: association of HPV-DNA and RNA-based methods. Int J Immunopathol Pharmacol 20: 341–347. 17624246 10.1177/039463200702000214

[38] Arbyn M, Roelens J, Cuschieri K, Cuzick J, Szarewski A, et al (2013) The APTIMA HPV assay versus the hybrid capture 2 test in triage of women with ASC-US or LSIL cervical cytology: A meta-analysis of the diagnostic accuracy. Int J Cancer 132: 101–108. 22610699 10.1002/ijc.27636

[39] Sørbye SW, Fismen S, Gutteberg T, Mortensen ES (2010) Triage of women with minor cervical lesions: data suggesting a “test and treat” approach for HPV E6/E7 mRNA testing. PLoS One 5: e12724. 20856930 10.1371/journal.pone.0012724 PMC2938337

[40] Szarewski A, Mesher D, Cadman L, Austin J, Ashdown-Barr L, et al (2012) Comparison of seven tests for high-grade cervical intraepithelial neoplasia in women with abnormal smears: the Predictors 2 study. J Clin Microbiol 50: 1867–1873. 22422852 10.1128/JCM.00181-12 PMC3372127

[41] Koliopoulos G, Chrelias C, Pappas A, Makridima S, Kountouris E, et al (2012) The diagnostic accuracy of two methods for E6&7 mRNA detection in women with minor cytological abnormalities. Acta Obstet Gynecol Scand 91: 794–801. 22486415 10.1111/j.1600-0412.2012.01414.x

[42] Solomon D, Davey D, Kurman R, Moriarty A, O’Connor D, et al (2002) Forum Group Members; Bethesda 2001 Workshop. The 2001 Bethesda System: terminology for reporting results of cervical cytology. JAMA 287: 2114–2119. 11966386 10.1001/jama.287.16.2114

[43] Moyer VA, on behalf of the U.S. Preventive Services Task Force (2012) Screening for Cervical Cancer: U.S. Preventive Services Task Force Recommendation Statement. Ann Intern Med. 2012 Mar 14.

[44] Ronco G, Biggeri A, Confortini M, Naldoni C, Segnan N, et al (2012) Health Technology Assessment Report: HPV DNA based primary screening for cervical cancer precursors. Epidemiol Prev 36(3–4 Suppl 1)1–72. 22828243

[45] Rijkaart DC, Heideman DA, Coupe VM, Brink AA, Verheijen RH, et al (2012) High-risk human papillomavirus (hrHPV) E6/E7 mRNA testing by PreTect HPV-Proofer for detection of cervical high-grade intraepithelial neoplasia and cancer among hrHPV DNA-positive women with normal cytology. J Clin Microbiol 50: 2390–2396. 22553244 10.1128/JCM.06587-11 PMC3405578

[46] Castle PE, Katki HA (2010) Benefits and risks of HPV testing in cervical cancer screening. Lancet Oncol 11: 214–215. 20089448 10.1016/S1470-2045(09)70385-7

[47] Sørbye SW, Arbyn M, Fismen S, Gutteberg TJ, Mortensen ES (2011) HPV E6/E7 mRNA testing is more specific than cytology in post-colposcopy follow-up of women with negative cervical biopsy. PLoS One 6: e26022, Epub Oct 6. 10.1371/journal.pone.0026022 PMC3188582 21998748

[48] Dillner J, Rebolj M, Birembaut P, Petry K-U, Szarewski A, et al (2008) Long term predictive values of cytology and human papillomavirus testing in cervical cancer screening: joint European cohort study. BMJ 337: a1754. 18852164 10.1136/bmj.a1754 PMC2658827

[49] Hariri J, Øster A (2007) The negative predictive value of p16INK4a to assess the outcome of cervical intraepithelial neoplasia 1 in the uterine cervix. Int J Gynecol Pathol 26: 223–228. 17581402 10.1097/01.pgp.0000236942.51840.56

[50] Ronco G, Giorgi Rossi P, Carozzi F, Confortini M, Dalla Palma P, et al (2010) Efficacy of human papillomavirus testing for the detection of invasive cervical cancers and cervical intraepithelial neoplasia: a randomised controlled trial. Lancet Oncol 11: 249–257. 20089449 10.1016/S1470-2045(09)70360-2

